# Enabling good outcomes in older adults on dialysis: a qualitative study

**DOI:** 10.1186/s12882-020-1695-1

**Published:** 2020-01-29

**Authors:** Rajesh Raj, Bridget Brown, Kiran Ahuja, Mai Frandsen, Matthew Jose

**Affiliations:** 1 School of Medicine, University of Tasmania, Hobart, Australia; 2 Launceston General Hospital, Launceston, Tasmania 7250 Australia; 3 School of Health Sciences, University of Tasmania, Hobart, Australia; 4 Faculty of Health, University of Tasmania, Hobart, Australia

**Keywords:** Elderly, ESKD, Dialysis, Outcomes, Lived experience, Quality of life, Qualitative research

## Abstract

**Background:**

Older patients on dialysis may not have optimal outcomes, particularly with regards to quality of life. Existing research is focused mainly on survival, with limited information about other outcomes. Such information can help in shared decision-making around dialysis initiation; it can also be used to improve outcomes in patients established on dialysis. We used qualitative research methods to explore patient perspectives regarding their experience and outcomes with dialysis.

**Methods:**

Semi-structured interviews with participants aged ≥70, receiving dialysis at a regional Australian hospital, were recorded and transcribed. From participants’ responses, we identified descriptive themes using a phenomenological approach, with verification by two researchers. Factors affecting outcomes were derived reflexively from these themes.

**Results:**

Seventeen interviews were analysed prior to saturation of themes. Participants (12 on haemodialysis, 5 on peritoneal dialysis) had spent an average of 4.3 years on dialysis. There were 11 males and 6 females, with mean age 76.2 years (range 70 to 83). Experiences of dialysis were described across four domains - the self, the body, effects on daily life and the influences of others; yielding themes of (i) responses to loss (of time, autonomy, previous life), (ii) responses to uncertainty (variable symptoms; unpredictable future; dependence on others), (iii) acceptance / adaptation (to life on dialysis; to ageing) and (iv) the role of relationships / support (family, friends and clinicians).

**Conclusions:**

Older patients experience the effects of dialysis across multiple domains in their lives. They endure feelings of loss and persistent uncertainty, but may also adapt successfully to their new circumstances, aided by the support they receive from family, health professionals and institutions. From these insights, we have suggested practical measures to improve outcomes in older patients**.**

## Background

By 2030, it is estimated that 4 to 7 million people will be receiving renal replacement therapy worldwide [1]. Those over the age of 75 make up 22% of all patients on dialysis in Australia [2], and figures are similar elsewhere (USA – 20%, UK - 16% and Japan - 31%) [3–5]. Elderly patients on dialysis, particularly if they have comorbidities, may not survive for long periods or have a good quality of life [6, 7]. This suggests that comprehensive conservative or supportive care, without dialysis, may be a valuable treatment pathway for such patients.

It is common for the older patient with advanced renal failure to frame the choice between dialysis therapy or conservative care as a choice between life (dialysis) or death (non-dialysis pathway) [8]. Clinicians can access several recommended tools to predict prognosis in elderly patients with advanced renal failure [9]. While these prognostic tools often consider survival, they may not predict quality of life or other outcomes, which may arguably be more important in these elders’ lives [10]. The intrusive nature of dialysis treatment alters multiple aspects of daily life, including the effects on physical and cognitive states, and the worsening of the complexities of ageing [11]. Information about these effects could be used by older adults choosing between dialysis and conservative care, or be used to suggest interventions to improve outcomes for patients already on dialysis [12].

In adapting to a major illness, the older patient is likely to have different priorities and coping strategies compared to their younger counterparts [13]. After starting dialysis some older patients appear to thrive, while others enter a progressive spiral of deterioration, dependency and repeated hospitalisation [14]. These qualitative and individual consequences of dialysis are hard to predict. In order to understand factors influencing these outcomes, distinct from survival or mortality, we considered that an exploration of the patient’s perspective would yield useful insights [15]. In this article, we report the results of a qualitative research study that used semi-structured interviews to document older patients’ experiences of dialysis and outcomes.

## Methods

### Study population, recruitment and sampling

A convenience sample of eligible participants was recruited from among patients under the care of a regional Australian hospital. Participants were eligible to participate if they were aged over 70 years and were being treated with haemodialysis (HD) or peritoneal dialysis (PD) for more than 3 months. Exclusion criteria included patients judged by their treating physician to be too unwell or cognitively impaired to participate; and patients unable to converse in English. The research protocol was approved by the Tasmanian Human Research Ethics Committee (H0014515).

Potential participants were invited to the study by the research nurse in person, provided information sheets and given the opportunity to read and ask questions of the study. Interested participants were then asked to sign a consent form. Face-to-face interviews were conducted at a time and place convenient to the participant. Interviews lasted approximately 45 min (range 30 to 75 min) and were audio recorded. Demographic data collected included age, gender, mode of treatment, years spent on dialysis, primary and secondary diagnoses (presented as the Charlson Comorbidity index) and laboratory parameters including haemoglobin, albumin, C-reactive protein, phosphate and weekly Kt/V reflecting dialysis adequacy for HD patients [16].

### Interviews

Questions for the semi-structured interviews were derived from informal email surveys of experienced nurses and nephrologists prior to starting the research project (Table 1; also see Additional file 1 in Supplementary files). The interviews were conducted face-to-face by a female registered nurse of 12 years’ nursing experience, trained in conducting qualitative research interviews. Only the research nurse and participant were present at the interviews. Most interviews (14 of 17) were conducted in participants’ homes; 3 interviews were conducted in a private room at the dialysis unit.

**Table 1:** Interview questions derived from an informal survey of doctors and nurses

Interview questions	Expected areas of interest for the study
1.How are you doing on dialysis, and why?	Patient perspective of current outcome and contributing factors
2. How do the people around you influence you - at home, or in the renal unit (doctors, nurses or other patients)?	Influence of family, friends, healthcare professionals or others on living with dialysis as an older person
3. How dependent / independent are you for: activities of daily living; other practical things (money, food, transport)? Who helps?	Exploration of the older patient’s dependence on / independence from social and institutional support
4. What are the best & worst things about life (on dialysis & overall)?	The older patient’s perspective of the salient aspects of life on dialysis
5. How do you see yourself if you were not on dialysis?	Exploration of the impact of dialysis on life course in the elderly
6. What are your thoughts regarding the future or advance care planning?	Patient perspectives of future outcomes and preparation for these

(The questions, recording equipment and transcription services were pilot-tested on a volunteer dialysis patient who was aged 52, and therefore not eligible for inclusion in the study).

### Data analysis

All interviews were audio-recorded and transcribed verbatim by external professional transcribing services (Outscribe Transcription Services, Australia). Transcripts were cross-checked for accuracy, and participants were offered the opportunity to read over their transcripts for accuracy (all declined). Transcribed interviews were then imported into data analysis software (NVivo qualitative data analysis Software; QSR International Pty Ltd., Australia. Version 10, 2014) to enable rigorous, low-error analysis of the transcribed text. The purpose of the analysis was to explore and describe the individual experiences and opinions of the participants with regards to dialysis treatment. A phenomenological approach was adopted, and iterative thematic analysis utilised to develop representative themes from the text.

Primary analysis of transcripts was conducted by author RR, utilising line-by-line coding to identify key concepts and issues. Codes / concepts were then grouped into themes and categorized. A memo and project log were kept throughout this process. Supplementary analysis of the data by author MF was conducted to verify themes and domains identified by RR [17]. Throughout this process there was reflexive consideration of the analysis and discussion between the two investigators [18, 19]. Key domains and major themes were identified via consensus of the authors. Interviews were conducted and analysed until there was saturation of themes, achieved after 17 participants were interviewed. Finally, potential predictive factors of outcome on dialysis were derived from the described experiences and themes by authors RR and MF (see Additional file 2 in Supplementary files).

## Results

Transcripts from 17 interviews were analysed prior to saturation of themes. Summary characteristics of the study population and the details of individual participants are presented in Tables 2 and 3 respectively.

**Table 2:** Summary characteristics of study population

Characteristic	Patient Data (*n* = 17)
Mean Age (SD)	76.2 (± 3.6)
Male / Female Gender (n)	11/6
Mean years on Dialysis (SD)	4.4 (± 2.5)
Mode of dialysis - HD/PD (n)	12/5
Mean Charlson’s Comorbidity Score (SD)	5 (± 2)
Mean Karnofsky Score (SD)	70 (± 10)
Diabetes (%)	35
Hypertension (%)	70
Ischaemic Heart Disease (%)	24
Peripheral Vascular Disease (%)	42
Mean Biochemical parameters (SD)	
Haemoglobin g/L	114 (± 10)
Albumin g/L	33 (± 3)
Phosphate mmol/L	1.57(± 0.25)
Kt/V Urea (those on haemodialysis)	1.30 (± 0.12)

**Table 3:** Individual Participant Characteristics

#	Age/Sex	Education	Cause of ESKD	CCM	Other Illnesses^a^	KPS	Years on dialysis	Modality	Interview duration^b^	Hb	Albumin	CRP	PO_4_	Kt/V
1	82/M	Year 4	Unknown	3	Gout	60	2	HD	45.36	105	38	3	1.54	1.29
2	75/M	Year 8	Chronic GN	3		80	4	HD	49.15	110	35	15	1.83	1.36
3	73/M	High school	Diabetes	7		60	9	HD	49.33	130	35	5	1.16	1.11
4	78/M	High school	Diabetes	7	Ulcerative Colitis	70	4	HD	51.51	110	37	6	1.48	1.24
5	75/M	High school	Hypertension	8		50	4	HD	60.20	107	32	11	1.82	1.56
6	71/M	Year 10	PAN	5	Deafness	70	4	PD/HD/Tx	40.40	138	33	15	1.78	–
7	75/F	High school	Hypertension	6	Hyperthyroidism, MD	60	8	HD/PD	37.25	116	24	26	1.89	1.16
8	80/F	Year 8	Hypertension	4	Aortic Stenosis	60	2	HD	36.47	128	34	8	1.87	1.32
9	78/M	High school	Hypertension	8		80	3	HD	61.30	112	35	4	1.26	1.25
10	70/F	Year 8	Diabetes	5		80	3	PD	49.49	121	32	7	1.32	–
11	83/M	None	Hypertension	4	Gout, diverticulitis	60	2	PD	36.38	109	32	5	1.6	–
12	79/F	Year 8	Hypertension	5		80	3	PD	37.20	107	36	4	1.3	–
13	75/F	High school	MSK	3		80	7	HD	42.32	112	33	9	1.82	1.33
14	75/F	Year 10	Diabetes	5		60	2	HD	39.21	105	31	13	1.57	1.25
15	78/M	Middle school	Hypertension	7	Parkinsonism	60	6	HD	36.40	111	35	4	1.17	1.26
16	78/M	High school	IgAN	3	Hypothyroidism, DVT	70	2	HD	52.13	107	32	7	1.56	1.46
17	74/M	High school	Hypertension	3	Coarctation of Aorta	80	9	PD	35.30	109	31	11	1.72	–

*M* Male, *F* Female, *ESKD* End-stage Kidney Disease, *CCM* Charlson’s Comorbidity Score [16], *KPS* Karnofsky Performance Scale [20], *Tx* Transplant, *Hb* Haemoglobin(g/L), *CRP* C-reactive protein, *PO*_*4*_ Phosphate, *MD* Macular degeneration, *DVT* Deep Vein Thrombosis, *IgAN* Immunoglobulin A nephropathy

^a^Other illnesses not included in Charlson’s Comorbidity Score; ^b^ Interview duration in minutes

Participants’ reports of their experience of dialysis and its effects could be classified under four main domains: (i) the concepts of self, (ii) the physical body, including symptoms, (iii) effects on everyday life and (iv) participants’ relationships with others. These descriptions are discussed in detail below.

### Dialysis and the self

“I don’t know, I’ve forgotten what it’s like not to be on dialysis”. [Female, 75, on HD after failure of PD]

Dialysis was described as a very intrusive treatment, which had a significant impact on the concept of self, caused major changes in lifestyle and altered the life-roles of most participants. For patients on peritoneal dialysis, there was frustration at having to go on the therapy every night, without significant breaks:

“Oh, I'd prefer to only do it, say, two or three nights a week, rather than doing it every night. For argument's sake, if you were in bed and wanted to jump out and run outside or run to the toilet or something you just hop out and go, don't you? You're not … I always say to my wife I'm tied up like a little dog in a kennel.” [Male, 74, discussing staying in bed overnight for PD]

There was acknowledgement that undergoing treatment was necessary to preserve life and the self. The decision to start dialysis and subsequently to continue it, despite discomfort, was framed as a choice between living and dying, since participants interpreted that without dialysis, death was certain. Even though dialysis was ‘chosen’ as a treatment option, there was ambiguity about whether there was choice after all, since there really was no other option if the person wanted to stay alive.

“…no well I’ve got, well I’ve got a choice. I can have dialysis or go up the chimney!” [Male, 75, on HD]

Being on dialysis altered how some participants thought about good outcomes in their lives. When replying to questions about how they were doing on dialysis, some participants tended to frame themselves in terms of their illnesses and responses to treatment. For instance, they said that overnight treatments or dialysis sessions without interruptions or medical problems indicated that they were having a good outcome. Similarly, ‘good’ biochemical test results, trouble-free machine behaviour, or positive reports from healthcare professionals implied positive outcomes for some, suggesting a shift from internal to external, ‘medicalized’ determinants of one’s status.

When asked about the reasons for their perceived good outcomes, participants cited their own personal factors. Prominent among these descriptions was the characterisation of themselves as independent entities that regained control over life’s events. Several participants described themselves as ‘stubborn’, ‘obstinate’ or ‘a fighter’. These narratives about control over life’s events was the most frequently coded theme. A personality that remained independent yet adapted to adversity and carried on was cited as a reason for doing well on dialysis.

“Yeah. I'm just one of those people that feel you've got to, you know, get on with life and get on, you know, if you've got a problem, just deal with it yourself”. [Male, 78, on HD]

Participants highlighted the importance of a positive attitude which enabled them to bear the difficulties of dialysis and ageing. Such an attitude was demonstrated in their refusal to worry about things, to take things in their stride, or in acknowledging that “the only one to help me is myself”. Participants spoke of never ‘thinking negative’. As one participant said, there’s “no point worrying it”.

“I don’t let anything worry me, and I take everything in my stride, I don’t go sulking to somebody about this or about that, I just put up with it all. And [my nurse] says, you’ve got a good attitude”. [Female, 75, on HD]

In response to questions about the future, the older age of participants in this study was reflected in their attitudes towards mortality and death. Several participants demonstrated a pragmatic approach; they acknowledged that life was limited, and some declared that they did not fear death.

“Just wait for the sun to rise the next day and we’ll live that one as it comes. That’s all you can say. Because we don’t know how long we’ve got…..I don’t think I’ve got that much left, really. Too bad to worry about it now”. [Male, 78 on HD]

Whereas all patients acknowledged the difficulties of living on dialysis treatment, most patients stated that they were doing well. They described adapting successfully and valued the lives they led on dialysis, despite all the difficulties. They accepted the necessity of dialysis treatment and adjusted their lives around the treatment, sometimes calling on family or healthcare personnel to help in their transition. For some such patients, stopping treatment and accepting eventual death was considered “giving up” of a valuable life.

“Oh, life’s too valuable to turn around and do a silly thing like that. Throw the sponge in like that, just get sick of it like that…” [Male, 73, on HD]

### Dialysis and the body

“Well, it’s hard to define because I have a problem – like, renal problem – right? And I also have an old age problem.” [Male, 82, on HD]

Our participants frequently mentioned the effects of persistent symptoms and progressive physical deterioration, compounded by the effects of growing older. This impacted on their ability to do things they had done earlier - a loss felt by several participants. Progressive loss of vision, worsening mobility and persistent fatigue were among the problems mentioned. Participants reported being now unable to read, drive or do things around the house unaided. Those on peritoneal dialysis also felt their physical restrictions acutely when they needed to handle their heavy bags of dialysis fluid.

“Just doing me housework and all that, you know. I used to do everything all at once, now I can't. Getting old.” [Female, 70, on PD]

Not all participants had relief from bodily symptoms after starting dialysis, and this led to contrasting perspectives regarding symptoms and their impact on the experience of dialysis. On the one hand, a subset of interviewees remembered being severely symptomatic with renal failure prior to beginning regular dialysis treatments and were grateful that starting therapy made them feel much better. They continued dialysis, despite its difficulties, because they did not want to once more feel as bad as they had prior to the initiation of dialysis.

“And I feel a lot better than what I did. But if you could have seen me before I got on dialysis, it was dreadful. Yeah.” [Female, 75, on HD]

On the other hand, there were others who developed a new set of symptoms as a result of the dialysis procedure itself - intolerance of fluid removal, the pain and uncertainty around inserting needles into the arteriovenous fistula (used for hemodialysis access) and the need to sometimes rest in bed for long periods after each dialysis session. The symptoms were unexpected for some patients, who had expected that dialysis would actually make them feel better.

“Well everybody tell me I’ll feel real good after it, but you don’t…No they told us that you know first off they said you’ll feel better and everything but you don’t.” [Male, 78, on HD]

A third group of participants had pre-existing bodily symptoms from other illnesses - such as low back pain, or diabetic complications - which did not improve, and even worsened the experience of dialysis. Patients troubled by worsening physical status, and a severe symptom burden, often stated that they were not doing well on dialysis.

### Dialysis and its effects on daily life

Participants reported multiple effects on daily life as a result of being on dialysis. The amount of time spent on the treatment was repeatedly mentioned. Several participants were frustrated with having to remain immobile for the 4 h or so of hemodialysis or the longer overnight periods of peritoneal dialysis. Along with the hours needed for treatment, those on hemodialysis also reported the time lost in travelling to and from the dialysis centre. In all, this left no time for other activities on dialysis days, especially if they felt unwell after dialysis and had to rest for a while afterwards.

The commitment to dialysis forced some to give up activities that they enjoyed, including travel, hobbies such as fishing, hunting or part-time work. Relatively inflexible dialysis schedules also meant that participants progressively withdrew from social engagements, thus significantly changing their social roles.

“But I’d spend, and I used to work behind the bar on a voluntary basis one night a week. There was always something to do and I’ve always got involved in things. And since I’ve been on dialysis … I had to give it away.” [Male, 78, on HD]

Several participants commented on the cyclical nature of symptoms related to hemodialysis treatments. Significant tiredness was common, especially if there had been large fluid removals during the session. This tiredness slowly improved until the next day, when they felt much better, only to reappear the following day after the next dialysis treatment. These repeating cycles of severe fatigue and relative wellness contributed to the intrusive nature of dialysis. It prevented participants from committing to activities outside of dialysis. It also made them increasingly dependent on external help, especially during the days of post-dialysis fatigue.

“...my kids used to say, ~You've got an extra day off. We can go off here, we can go there... but you don't, those days is when you feel like you want to have a bit of a rest or something because, as I said, you feel, you don't feel like you're full of bean.” [Male, 82, on HD]

This episodic nature of symptoms was not prominent for patients on peritoneal dialysis. However, some patients described feeling more energy and better concentration in the mornings rather than later during the day.

The food and fluid restrictions imposed impacted participants’ daily lives and their social interactions. Fluid restriction was difficult for some; participants had to be conscious about these restrictions all the time, particularly when eating outside the home (including when eating with family or friends). Others felt that fluid restriction contributed to symptoms such as constipation and this prompted them to be non-compliant.

“But it’s hard, because I’ve been … with clubs and things like that, and to go and have a drink and a cup of tea and, so now I’m not allowed to have it.” [Male, 75, on HD]

A few participants understood the necessity of restricting fluids, and made necessary adjustments, believing that their adherence to fluid restriction enabled them to do well on dialysis. Families and friends also contributed to helping patients maintain their restricted diets. Peritoneal dialysis patients with preserved urine output did not report difficulties with fluid restriction.

Most of our participants were retired from work. One of them had his own business but reported that the time commitments of dialysis had forced him to hand over responsibility for everyday matters to others. Those who did not have financial stability reported difficulties with the increased expenses. This was particularly true for those that lived far from the dialysis unit if they had to pay transport charges. Some participants had to move homes to be nearer to the units, once again interfering with social connections.

### Dialysis and others

In this population of dialysis patients over the age of 70, relationships with others - partners, other family members, friends, neighbours and healthcare professionals - were important in how they experienced life on dialysis.

The presence of a loving spouse or family members appeared to influence the decision to start and continue dialysis. As our cohort experienced increasing loss of physical abilities, people around them helped them cope. Many received help with activities in the house, including help with therapy for some patients on peritoneal dialysis. Others were assisted with chores such as tending to the garden or shopping at supermarkets. This support structure of family and friends had positive influences on how they coped with dialysis.

Friends and neighbours were also relevant to most participants, for both the practical benefits in and around the house as well as the psychological benefits of interacting and staying in touch. Some participants considered it important to maintain relationships not connected to their life on dialysis. Other participants mentioned that over time the staff and other patients and families at the dialysis became part of an extended group that they could relate to. Dialysis units provided an environment to meet more people. The shared experiences of dialysis, including the many restrictions, the long hours spent together at the unit and the similar interactions with healthcare personnel, strengthened these bonds. The camaraderie and humour lifted spirits.

“Yeah, I think so, it helps you with your, you talk about, well some will moan and groan about things, and some will just talk like happy-go-lucky, and just forget their illness, talk about other things…” [Female, 80, on HD]

Our participants all had close relationships with nurses, and acknowledged the central role played by nurses in their lives. For patients on haemodialysis, the nurse looking after them on the day had a significant impact - both on the conduct of dialysis (including needling of the AV fistula) as well as through how they made the participants feel. PD patients recalled the training and support they received from nurses, and acknowledged the important roles played by the nurses in their successful conduct of PD. Both positive and negative interactions with nurses were recounted. When participants perceived a lack of interest in their welfare, they were impacted adversely. Nurses that took an interest in their patients and spoke kindly were appreciated. Overall, most participants appeared grateful for the care they received from their nurses and considered them part of a “new family”; there were several anecdotes of humorous interactions. Nurses often instruct patients on food and fluid restrictions or peritoneal dialysis technique - participants had varied reactions to this. While some appreciated the advice, others were not happy being told what to do.

“Yeah, I mean you know they say oh he’s not supposed to do this, not supposed to – hang on a minute, I’ve got to have some, bloody quality of life. I’m not going to just starve myself.” [Male, 73, on HD]

Most participants acknowledged the essential role played by doctors and trusted them implicitly to look out for the welfare of their patients. It was important to get along well with doctors. Participants emphasized how valuable it was to them that doctors considered them as individuals and showed respect and involvement. A sense of humour was appreciated. Tone of voice, manner of speaking and consistency of behaviour were important too.

“…but just his approach to the patient and everything like that, always ready to listen and smile on his face.”[ Male, 75, on HD]

Negative interactions with doctors had a significant impact on the participants. Some participants felt let down by doctors who did not interact well, and preferred health professionals that they could better relate to.

“…like every six weeks I’m supposed to come, and you talk to the nurse and don’t talk to me?…[made me feel] that I was inferior, that he thought he was too good to talk to me, do you know what I mean....but this other doctor has been different.” [P6, Female, 75, on HD, discussing clinic visits to her specialists]

Our older participants relied on health care professionals for most of their medical information. Some participants were involved in their own care and were enthusiastic about asking questions to understand their treatment or the working of the dialysis machine. For others, there was no desire to gather more information and instead they relied on professionals “knowing what they are doing”.

Interestingly, some participants felt doctors could not help because doctors were too busy, or that they had not experienced first-hand what patients had gone through. This difference was highlighted by a participant who stated:

“He [the doctor] hasn’t fallen on the floor, he hasn’t, and carted him off to hospital so he’s okay, you know. So, as far as I’m concerned it is a waste (to speak to doctors).” [Male, 78, on HD]

These beliefs eroded their trust in the doctor-patient interaction; they stated that there was no benefit in meeting their doctors regularly. Such comments were common when they felt that medical professionals had not been attentive enough or had not communicated well enough to satisfy their expectations.

Several participants commented that the experience of dialysis was quite different to what they had expected, or that they had not been given enough information.

“They just plonk you on the machine and that's it, you know, they do it, and they didn't explain things.” [Female, 75, on HD]

Even participants who had received formal, structured pre-dialysis education regarding dialysis treatment did not retain all of the information received. (We could not explore, within the limitations of our design, if the participants had deficits in learning, memory or other aspects of cognition, or whether the methods of patient education locally available were unsuitable for this older cohort.) Patients who switched from one form of treatment to another, or returned to a therapy after a failed transplant, were much better prepared.

## Discussion 

Our findings reflected the intrusive nature of dialysis, which impacted on almost every aspect of the life of the older adult on this treatment. There were four main overlapping meta-themes spread across domains: loss, uncertainty, acceptance and support. (Table 4).

**Table 4:** Themes arranged according to domains

	Loss	Uncertainty	Acceptance	Support/ Relationships
Dialysis and the self	- Of choice: it is now either dialysis or death - Of control: nothing can be done about it - Of identity; personhood: dialysis must go well for me to be okay - Of pre-dialysis life: role, activities, ideas for the retired life	- Dialysis sessions (determine how I feel) are unpredictable - The machine tells you how I am doing, not me. - Rely on HCPs to communicate clearly: otherwise, I know nothing. - No future hopes, other than to continue dialysis until death.	- Rationalizing the need to be on dialysis - Positive outlook - Taking control of life - Use of humour to cope - Life is worth living, purposeful	- Relationships are crucial: as support and as reason for living - HCP interactions are crucial - HCPs cannot do much if they do not know how I live
Dialysis and the body	- Of the sense of “normality”: now the machine-led life. - Of wellbeing: the prominent symptoms during and after dialysis - Of health: other medical issues continue - Of physical and mental functions through ageing	- About needling of AV fistula - pain, bleeding - Unpredictable symptoms caused by dialysis - Fluid removal on dialysis and its effects: on energy, on BP - Discomfort in the dialysis unit- chairs, temperature - Other persistent symptoms - Other unexpected illnesses, including the fear of peritonitis - Thoughts about mortality	- Acknowledge effects of ageing - Ask for help when needed - Symptoms relieved by dialysis - Pragmatic discussions about death and functional limitations - Participation in advance care planning, including options for dialysis withdrawal	- Receiving help to look after oneself - Discussion of advance care plans with family, HCPs - Discussing health issues with HCPs
Dialysis and daily life	- Of time: for everyday things; social activities - Of dietary choices: fluid and food restrictions - Of travel possibilities: all trips linked to dialysis services - Of finances: transport costs, phone bills, lost earnings	- Repeating cycle of wellness and fatigue around the days of HD - Episodic nature of HD: the need to arrange life around dialysis times - What is done on a day depends on how the dialysis session went.	- Choosing activities according to situation & capability - Optimising health to engage in preferred activities - Seeking help where needed - Accepting and modifying diet/intake	- Receiving support from HCPs/ allied health - Maintaining and strengthening helpful relationships among family and friends - Making time for social activities
Dialysis and others	- Of social ties - Of agency: the new need to comply with HCP instructions, rules for dialysis patients	- Others did not communicate: dialysis is not how I expected - Social commitments now depend on dialysis schedules	- Accepting help where available - Choosing to adhere to HCP recommendations	- Engagement with HCPs to improve the experience of dialysis - Maintain activities / relationships outside dialysis - Family / friends / relationships that are nurturing - Dialysis unit as a new family or social outlet

### Loss

Our population of older patients on dialysis felt a pervasive sense of loss across all the four domains of experience. Lindquist speaks of the dialysis patient’s wishes for independence and normality - both these subjective feelings are lost when on dialysis [17]. Participants reported significant changes to their lives after starting dialysis, similar to the feeling of “life being lost” described by Monaro and colleagues [18]. Changes in participants’ concepts of themselves were seen when they described their health in terms of machine performance or biochemical targets. Various authors have described this as a transition into a life restricted [19]; not finding space for “living” [21] or a life with physical shackles [22].

McDonald described a continuum in responses to a life on dialysis, one aspect of which is a struggle between control and acquiescence [23]. Similar to this, in our population, some participants felt disempowered by their losses, while others learnt to adapt to them and carry on. Such participants described positive adaptation and the transformation to a new self, capable of dealing with the new realities, and appeared to have good outcomes on dialysis (see discussion below).

### Uncertainty

Uncertainty is another concept that spans several domains in our results. The repeated cycles of tiredness and improvement coinciding with HD sessions three times a week meant our participants were never sure of how they would feel, since dialysis sessions determined their status (variables such as large fluid removal targets, problematic needle insertion into the arteriovenous fistula or the behaviour of the dialysis nurses or doctors on the day). Those undergoing PD had a persistent fear of peritonitis and its effects. These feelings of vulnerability and uncertainty have been highlighted in several similar studies in the literature [21, 24].

The future was unclear; several participants acknowledged that longevity was not certain and were happy to discuss advance health care directives. The inadequacies appearing with ageing and the experience of other dialysis patients (or other older acquaintances) dying impacted on their own outlook for the future. This uncertainty introduced by dialysis could worsen the tendency of older adults to have lower “meaning in life” scores [25].

### Acceptance/adaptation

Several researchers have studied the process of ageing, prominent in the narrative of our participants, as a process of adaptation to declining physical and cognitive capabilities [26, 27]. Previous studies have identified the theme of “attempting to maintain manageability” as part of life on dialysis [21]. We identified adaptations in our older population as a series of changes - in lifestyle, activities, diet, fluid intake and mental attitude, undertaken with the aim of optimizing outcomes. It appeared that our participants on PD reported fewer problems adapting to treatment. In general, patients who tended to do well adapted to dialysis in positive ways, seeking to optimize their lives and in this way, to maximize the benefits of the restricted life on dialysis. Rittman describes this attempt by patients on dialysis to retain control over their lives by negotiating a new understanding of life and maintaining hope [28]. This contrasted with those reports where patients did not show this acceptance, and instead focused on the difficulties. These patients did not engage in making adaptations to life in order to deal with adversity [29, 30].

### Relationships and support

Family members (and/or friends) had significant impacts on how life was perceived. Some participants felt that the involvement of their family was responsible for their positive experience of dialysis; on the other hand, others stated that they stayed on dialysis in order to take care of their family members. Interactions with family - including the new acquaintances at the dialysis unit - were related to “meaning in life” and to hope for the future. This is consistent with other studies which describe the ageing patient attempting to regain control of their life roles as their care situation or dependency needs change [31].

Nurses played an important role in the lives of these patients. Their skills and their interactions with patients determined how dialysis was perceived. Similar observations have been reported by Madar, who commented that nurses have significant impacts on reducing the stress of dialysis [32].

With regard to the relationship with doctors, the need to be seen as “normal” “as a human being”, and “with respect” was manifest, similar to other qualitative studies [21, 31]. There was a spectrum of variable expectations from older patients on dialysis, emphasizing the importance of an individualized approach based on patients’ needs.

Our study had limitations. The limited number of participants may not be representative of the entire population. We interviewed participants already on dialysis, who were not cognitively impaired, and this could have biased results, since cognitively intact older patients who are on active treatment may represent a cohort more likely to achieve good outcomes. We did not design our study to compare HD and PD modalities, but it is likely that there are differences in outcomes between them; similarly, there may be differences in the cohorts that choose each form of treatment. However, we have focused on the overall impacts of dialysis treatment and believe that our insights address issues common to all forms of treatment undertaken by the older individual, who contends with declining abilities, increasing dependency and uncertain outcomes.

## Conclusions: our results in the context of clinical practice

The predictors of a good outcome on dialysis, listed in Table 5, were postulated by reflexive analysis, drawing interpretations from participants’ descriptions of positive and negative experiences on dialysis. Most of these factors can be assessed using targeted history-taking, or using several validated questionnaires and other tools, some of which are listed in Table 6. When indicated, some psychological factors may be amenable to interventions such as cognitive behavioural therapy. Our research also highlights several social factors which have an influence on dialysis outcomes, thus highlighting the importance of a holistic approach to the elderly person considering dialysis.

**Table 5:** Predictors of a good outcome and methods of assessment, derived from reflexive interpretative analysis

Physical factors	
● prominent uraemic symptoms that may be relieved by dialysis (e.g., nausea, anorexia)	
● low levels of pre-existing frailty/physical dependence	
● absence of pre-existing significant symptoms that are unlikely to be relieved by dialysis (e.g., chronic pain, depression)	
● the ability to tolerate dialysis, particularly fluid removal	
● a functional access for dialysis which is not problematic to use/maintain	
Psychological factors	
● lack of conflict or ambiguity around the decision to start dialysis	
● expectations from dialysis that are reasonable and achievable	
● illness perception - an internal locus of control, willingness to take responsibility for own health	
● understanding of dialysis treatment and need for lifestyle changes, food/fluid restrictions	
● actively choosing a positive attitude; not “giving up”, willingness and opportunity to adapt to changing circumstances	
● hopeful; engaged with the future; “meaning and purpose” in life	
Social factors	
● family as motivation: providing physical/psychological support, family that requests continuance on dialysis or other treatment, participants who continue dialysis in order to be able to look after their family members	
● involvement of close family/friends/carers in daily life, in healthcare decisions	
● participants who derive social benefit from interactions of the dialysis unit (particularly if socially isolated)	
● ability to travel or engage in other activities (personal or social) separate from dialysis	
Healthcare provider/institutional/societal factors:	
● positive relationships with healthcare providers, where patients feel valued and listened to	
● appropriate skill sets among medical and nursing staff	
● opportunity to consider or participate in advance care planning	
● patient-friendly staff and dialysis facilities (e.g., flexible schedules, comfortable chairs, adequate heating)	
● easy access to dialysis facilities, including proximity, transport arrangements	
● financial stability or lack of financial penalties from being on dialysis	
● access to social/formal community support that is affordable and always available

**Table 6:** Some interventions to improve dialysis outcomes

Factors potentially leading to poor outcomes	Suggested Interventions	Objective Assessment (clinical / research purposes)
The decision to have dialysis framed as a choice as between dialysis (living) versus dying; decisional conflict	Specific discussions around choice; presentation of alternatives to dialysis such as maximal supportive care; involvement of family / carers in decision-making	Decision support aids (e.g., web-based aids, [33]
		The Yorkshire Dialysis Decision Aid (YODDA) [34])
		The ‘SURE’ test [35]
Undue expectations of symptom benefit from dialysis	Discuss inconsistency of symptom relief; appearance of new symptoms with dialysis (e.g., needling pain, fatigue)	Symptoms /quality of life surveys [36]
		Frailty indices [37]
		Comprehensive Geriatric Assessments (CGA) [38]
Being ill-prepared for the restrictions and the reality of life on dialysis	Information tailored for the older patient (more time, more repetition); Specifically discuss restrictions to travel, diet, fluid intake	Assessment of health literacy [39]
		Becker-Maiman model for analysis of compliance [40]
		Beliefs and Behaviour Questionnaire (BBQ) [41]
		Dialysis Diet and Fluid non-adherence Questionnaire [42]; Illness perception questionnaire [43]
Effects of ageing, physical or cognitive decline	Screen for frailty and risk of falls; prevent deterioration if possible, address frailty, monitor functional status, provide support before the patient “fails”	
Curtailment of activities outside dialysis; changing life-role	Explore personal values, discuss impacts of dialysis on the rest of the patient’s life	
The time commitment; losing time for ‘living’	Specifically discuss time lost - including time needed for travel, and the time lost resting after dialysis.	
Impact and recurring nature of post-dialysis fatigue	Warn patients of cyclic nature of symptoms like post-dialysis tiredness and their impact on life	Dedicated fatigue scales / inventory [44]
Lack of a “positive attitude”, actively adapting to effects of dialysis on life	Clinician focus and involvement in facilitating psychological adaptation, consider behavioural therapy if needed	Illness perception questionnaire [43]
		Inventory of Coping Strategies Used by the Elderly ICSUE [45]
Inability to maintain or enjoy goals /values / activities outside of dialysis	Encourage and plan with patients regarding: Selecting the right activities according to current limitations, optimising self for their performance, and making compensations / accepting help where needed	Life Attitudes Profile [46]
		Personal Meaning Profile [47]
Loss of the feelings of being valued, loved, supported.	Focus on meaningful clinician interactions; monitor support from family, friends; Consider needs of carers.	Quality of life scales [36]
		Trust in Physician Scale [48]
		Zarit Burden Interview [49]

Our analysis raised the possibility that the experience of life on dialysis was different for patients on PD compared to those on HD. Our study was not designed to specifically study this difference, but it is possible that the mode of therapy and the place of therapy (home versus centre-based treatment) could have significant effects. Those on PD were quite concerned with the smooth conduct of therapy and often commented on significant interactions with PD nurses. As they became weaker physically, they relied significantly on family members as they still needed to conduct their therapy by themselves at home. Other than the resentment at being confined to bed for long hours every night, in the limited number of patients on PD in our study, there were fewer reported difficulties with adapting to dialysis. We suggest that the direct comparison of HD and PD in the elderly population with regard to their impacts on outcome are an important area for future research.

Table 6 offers some practical suggestions to mitigate poor outcomes on dialysis for the elderly and provides a list of objective assessment tools that may be useful. Nephrology teams may not possess all the skills required to ensure good dialysis outcomes for the elderly, and a multidisciplinary approach, with involvement of other specialists, including geriatricians, psychologists, nurse educators and social workers may be optimal.

The realities of ageing and its associated problems continue for patients on dialysis, as do the difficulties caused by other comorbid conditions. Patients reporting good outcomes have often modified their activities according to capability, accepted support from those around them, and sustained beneficial social ties. An active choice to undertake dialysis treatment, with awareness of the difficulties of life on dialysis and in an environment of adequate support will increase the chances of being able to adapt successfully and experience good outcomes.

## Supplementary information

**Additional file 1.** Participant Interview Guide.

**Additional file 2.** COREQ checklist.

## Data Availability

Transcripts of interviews are stored in electronic format on secure servers at the University of Tasmania. All data generated during analysis in the study are also stored on these servers; where deemed necessary for publication, they have been included in this published article.

## Abbreviations

HD: Haemodialysis
Kt/V: Stands for Kt/V_urea_, which is the clearance of urea multiplied by dialysis duration and normalized for urea distribution volume; used as a surrogate for dialysis adequacy
PD: Peritoneal Dialysis
