# Supplementary material for: Enabling good outcomes in older adults on dialysis: a qualitative study
Source: BMC Nephrol. 2020 Jan 29;21:28. doi: 10.1186/s12882-020-1695-1 (PMC6988330; doi:10.1186/s12882-020-1695-1)
Supplement: Supplementary file 1 — Additional file 1. Participant Interview Guide. [file 12882_2020_1695_MOESM1_ESM.pdf]

Participant interview guide

1. **Introduction**
    a. Introduce yourself.
    b. Explain purpose of study; how interview is to be conducted and analysed.
    c. Offer option to review and edit interview after recording or transcription.

2. **Participants' perception of outcome**
    a. How are you doing on dialysis? (Are you doing well or poorly?)
    b. Why do you think you are doing that way? (Explore what the participant's thoughts on what affecting the outcomes on dialysis).

3. **The experience of being on dialysis**
    a. What are the best and worst things about life, in general, for you now?
    b. What are the best and worst things for you about life on dialysis?
    c. If you were not on dialysis, what would you be doing?

4. **Relationships**
    a. How do the people around you influence how you cope with dialysis? (Role of family, friends, social networks)
    b. Tell me about your relationships with nurses / doctors. (How do you get along? Can you discuss all your concerns with them? What role do they play / how do they help?)

5. **Managing life on dialysis**
    a. Regarding practical things (activities of daily living, money, transport, food) – how much do you still do?
    b. How much do others help? Who helps?

6. **Thoughts of the future**
    a. How do you see the future playing out?
    b. Have you thought about treatment options if you were to be seriously unwell? (explore thoughts regarding advance care planning, healthcare proxies)
