# Supplementary material for: Enabling good outcomes in older adults on dialysis: a qualitative study
Source: BMC Nephrol. 2020 Jan 29;21:28. doi: 10.1186/s12882-020-1695-1 (PMC6988330; doi:10.1186/s12882-020-1695-1)
Supplement: Supplementary file 2 — Additional file 2. COREQ checklist. [file 12882_2020_1695_MOESM2_ESM.pdf]

Consolidated criteria for reporting qualitative studies (COREQ): 32-item checklist

No.	Item	Comments
**Domain 1: Research team and reflexivity**		
1	Interviewer/facilitator	BB
2	Credentials	**BB** *-Masters in Public Health; BN; Cert. in Neph. Nursing;* **RR** - *DM, MRCP(UK), FRACP, PhD candidate;* **MF** – PhD (Psychology); **KA** – MSc Clinical Trials, PhD; **MJ** – FRACP, FASN, PhD
3	Occupation	**BB**-Nurse; **RR,MJ**- Nephrologist; **MF**-Qualitative researcher; **KA**- Research Scientist & Educator,UTAS
4	Gender	BB- *Female*
5	Experience and training	**BB** (interviewer) – Formally trained in qualitative research, interviewing; **RR** (primary author and analysis) – Trained in qualitative research
6	Relationship established	Some participants known to BB, RR
7	Participant knowledge of the interviewer	**BB** is a nurse with experience in dialysis<br>**RR** is a Nephrologist undertaking research towards his PhD
8	Interviewer characteristics	**BB** is a nephrology nurse with interest in pre-dialysis education and supportive care; **RR** is a nephrologist with interest in outcomes in the elderly with renal failure; and is a PhD candidate at the University of Tasmania
**Domain 2: study design**		
9	Methodological orientation and Theory	Phenomenology; iterative thematic analysis
10	Sampling	Convenience sampling
11	Method of approach	Face-to-face
12	Sample size	n=17
13	Non-participation(reason)	n=1; “not interested”
14	Setting of data collection	Participant’s homes (n=14) or dialysis unit (n=3)
15	Presence of non-participants	No
16	Description of sample	Participants aged ≥70 years (range 70–83); undergoing dialysis in the period 2014-2016; all Caucasian, English-speaking; 11 males, 6 females; none were employed
17	Interview guide	Attached. Yes, pilot tested.
18	Repeat interviews	No.
19	Audio/visual recording	Yes. Audio recordings.
20	Field notes	Yes.
21	Duration	45 minutes (range 30 to 75 minutes)
22	Data saturation	Yes
23	Transcripts returned	Participant offered chance to review; none requested this.
**Domain 3: analysis and findings**		
24	Number of data coders	RR/BB/MF
25	Description of coding tree	No
26	Derivation of themes	Derived from data
27	Software	NVivo qualitative data analysis Software; QSR International Pty Ltd., Australia. Version 10, 2014
28	Participant checking	No. Participant offered chance to check; none requested this.
29	Quotations presented	Yes; identified by anonymised participant numbers

**30**	Data and findings consistent	Quotations provided to illustrate each theme
**31**	Clarity of major themes	Yes; identified as metathemes (Table 2)
**32**	Clarity of minor themes	Yes; see text under ‘Results’ and Table 2
